# Disrupting future discounting: a commentary on an underutilised psychological approach for improving adherence to diet and physical activity interventions

**DOI:** 10.1017/S136898002200252X

**Published:** 2023-05

**Authors:** Naomi Kakoschke, David N Cox, Jillian Ryan, Ian Gwilt, Aaron Davis, Paul Jansons, Barbora de Courten, Grant Brinkworth

**Affiliations:** 1 Human Health, CSIRO Health & Biosecurity, SAHMRI, North Terrace, Adelaide 5000, Australia; 2 BVA BDRC, Sydney, Australia; 3 UniSA Creative, The University of South Australia, Adelaide, Australia; 4 School of Exercise and Nutrition Sciences, Deakin University, Melbourne, Australia; 5 Health and Biomedical Sciences, RMIT, Melbourne, Australia

**Keywords:** Future discounting, Non-communicable disease risk, Lifestyle behavioural interventions, Episodic future thinking

## Abstract

Non-communicable diseases (NCD) such as CVD and type 2 diabetes mellitus are major contributors to the burden of disease. NCD are largely driven by modifiable lifestyle factors including poor diet and insufficient physical activity, and consequently, prevention is a public health priority. Although diet and physical activity levels can be improved via lifestyle interventions, long-term adherence to such interventions remains low, which limits their effectiveness. Thus, it is critical to identify the underlying mechanisms that challenge uptake and adherence to such interventions. The current commentary discusses an important, but underexplored, psychological driver of poor adherence to lifestyle interventions, namely, future discounting, which describes the tendency to prefer smaller, short-term rewards over larger, long-term rewards. For example, in the nutrition domain, future discounting refers to valuing the immediate reward of excessive intake of energy-dense, nutrient-poor, discretionary foods high in salt, sugar, and saturated fat, and insufficient intake of low-energy, nutrient-dense, whole foods such as vegetables. Prominent theoretical models propose that excessive future discounting is a major contributor to the development of unhealthy lifestyle behaviours. Furthermore, a vast body of evidence suggests that future discounting plays a key role in risk of NCD. Thus, the evidence to date supports the idea that future discounting is an important multi-behaviour target for supporting lifestyle behaviour change; however, this approach has been largely neglected in preventive health efforts. Furthermore, this commentary discusses promising techniques (e.g. Episodic Future Thinking) for disrupting future discounting to promote improved adherence to lifestyle interventions aimed at reducing NCD risk.

Non-communicable diseases (NCD) including CVD, some cancers, respiratory diseases and type 2 diabetes mellitus (T2DM), are the leading cause of 71 % of deaths globally each year and major contributors to the burden of disease, illness and disability^(1)^. NCD are largely driven by modifiable lifestyle factors including poor diet (i.e. excessive energy intake and high intake of discretionary foods) and physical inactivity^(2)^. Consequently, preventive health is a public health priority, including the development of lifestyle interventions aimed at improving diet and reducing physical inactivity^(3,4)^. Long-term intervention studies and meta-analyses have consistently shown that consuming a diet comprising low-energy, nutrient-dense whole foods, increasing physical activity (PA) and reducing sedentary behaviour reduces NCD risk factors^(5,6)^. A recent Delphi study involving health professionals and individuals at risk of T2DM identified key intervention targets including PA, diet and mental health^(7)^. Unfortunately, adherence to lifestyle interventions remains a widespread problematic issue, particularly over the long term, which limits the effectiveness of these interventions^(8)^. For example, the WHO have reported that 67 % of patients with T2DM do not increase their level of PA after being diagnosed^(9)^. Consequently, there is a critical need to identify and disrupt factors that interfere with adherence to lifestyle health behaviour changes.

The aim of this commentary is to discuss an important, but underexplored, psychological driver of poor adherence to lifestyle behaviour interventions, namely, future discounting. Future discounting is a facet of impulsive decision-making that refers to the extent to which future rewards are reduced in subjective value as a function of delayed receipt^(10)^. A higher future discounting rate has been linked to chronic health issues such as substance use disorders^(11)^ and obesity^(12,13)^. Importantly, as future discounting is modifiable, it represents a prime intervention target^(14)^. Here, we discuss promising techniques (e.g. episodic future thinking, EFT) for disrupting future discounting to promote improved adherence to lifestyle interventions to reduce risk of NCD.

## What is future discounting?

Challenges in uptake and adherence to lifestyle interventions are underpinned by the human psychological tendency for future discounting, which is grounded in evolutionary biology^(15)^. Future discounting describes the tendency to prefer smaller, short-term rewards over larger, long-term rewards^(16)^. Future-oriented thinking plays a critical role in the ability to prioritise future goals over immediate discomfort (e.g. going for a run on a cold morning to maintain physical fitness in the long term) and short-term pleasure (e.g. an enjoyable sedentary activity such as watching TV which is detrimental to health in the long term). For millennia, our biology has led us to seek out energy-dense foods in an environment of scarcity^(17)^. Consequently, humans have developed an innate preference for consuming energy-dense foods, identified as those with fatty mouthfeel, salty and sweet taste sensory properties as humans find them immediately rewarding^(18)^. However, today, we live in a continuous feast, and excessive consumption of widely available highly processed discretionary foods (high in fat, salt or sugar) is contributing to an increasing prevalence of overweight, obesity and diet-related chronic diseases^(19)^.

Whilst the discounting of long-term rewards is a somewhat unanimous phenomena, excessive discounting is posited to be an important psychological mechanism underlying multiple disorders and unhealthy behaviours^(20)^. In the health domain, future discounting refers to valuing immediate rewards of excessive intake of energy-dense, nutrient-poor, discretionary foods high in salt, sugar, and saturated fat because they taste better, and insufficient intake of low-energy, nutrient-dense, whole foods such as vegetables^(21,22)^. A vast body of evidence suggests that future discounting plays a key role in NCD risk. For example, data from the Human Connectome project showed future discounting was the strongest of twenty neurocognitive predictors of obesity^(23)^. In a large Australian sample, future discounting was a significant predictor of both pre-diabetes and T2DM prevalence^(24)^. Recent work indicates that future discounting is a behavioural indicator of an imbalance between reward and executive function systems^(25)^. Furthermore, a recent review of 153 studies showed that future discounting of long-term future rewards was a key predictor of poor diet, low PA levels and weight gain, particularly in people with low socio-economic status^(26)^. Thus, the evidence to date supports the idea that future discounting is an important multi-behaviour target for supporting lifestyle behaviour change; however, this approach has been neglected in preventive health efforts.

## How does disrupting future discounting benefit future health?

The psychological drivers that encourage immediate gratification, such as consuming energy-dense, nutrient-poor discretionary foods or remaining sedentary, are deep-seated and stem from our evolutionary roots. Prominent models of temporal discounting from economics include the hyperbolic/quasi-hyperbolic discounting models^(14)^. The ‘hyperbolic’ model posits that the discounting rate is hyperbolic rather than exponential, that is, people are more impulsive towards sooner rewards and more patient for later rewards, often referred to as a ‘preference reversal’, which is represented by one discount rate parameter^(27)^. The quasi-hyperbolic model describes the difference in preferences for immediate and future rewards using two parameters; however, these models have been critiqued for their lack of predictive power^(27)^. In contrast, neuroscience models incorporating the role of emotion in future discounting have become more prominent^(28)^. One such model that explains the role of future discounting in health-related decision-making is the Competing Neurobehavioral Decisions System Theory (CNDS)^(29)^. The CDNS describes a dual-system decision-making conceptualisation of the psychological processes involved in making healthy choices. Specifically, the CDNS model posits that two competing neurobiological systems drive health behaviour. On the one hand, the reward-impulsive system is driven by limbic and paralimbic brain regions involved in undesired, risky consequences, while on the other hand, the evolutionary newer executive system is driven by prefrontal and parietal brain regions involved in self-regulation of behaviour^(29)^. Thus, the CDNS provides a framework for describing the combination of neurological and behavioural processes that account for future discounting and has formed the basis for psychological interventions aimed at modifying future discounting by modulating emotion^(14)^.

**Fig. 1:**
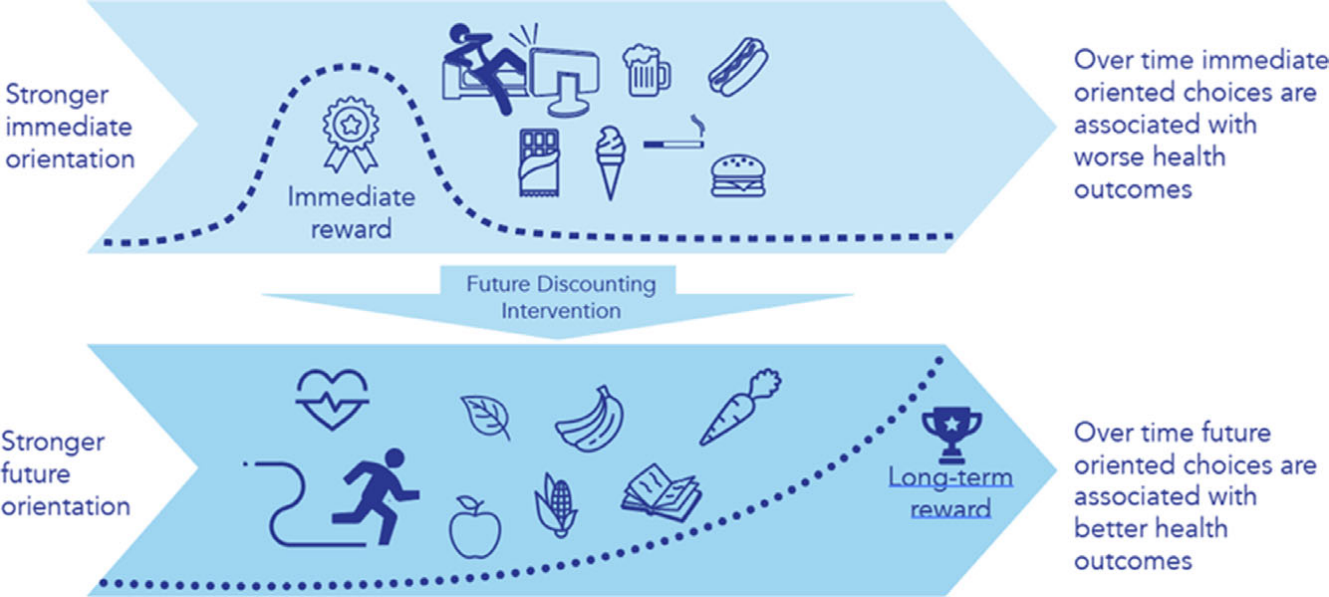
The hypothesised effect of domain-specific episodic future thinking on future discounting

Indeed, empirical evidence suggests that the rational brain can be trained to overcome innate and ‘affective’ urges. Promising interventions, based on psychological training techniques, have disrupted future discounting related to maladaptive health behaviours in numerous pilot studies^(30,31)^. Specifically, psychoeducation can assist individuals to understand the phenomena of future discounting, while cognitive behavioural training techniques can teach individuals to recognise feelings of instinctual hunger and provide strategies for overcoming urges to make unhealthy choices, especially at vulnerable moments (e.g. when individuals are hungry, tired or have limited available options). Several types of cognitive training techniques have been successfully used to achieve future-oriented thinking. One commonly used cognitive training technique is EFT, a type of mental prospection, involving the ability to vividly imagine and pre-experience possible future scenarios^(32,33)^. EFT uses psychoeducation and cognitive behavioural techniques to shift reward orientations from the immediate towards the future. Moreover, EFT involves individuals generating vivid, meaningful future scenarios to ‘project oneself into the future’ driven by episodic memory^(34)^. For example, EFT prompts individuals to imagine positive, realistic, personally relevant events that may occur at future time points (e.g. birthdays or holidays occurring periodically), whilst also identifying realistic, important and specific health goals. Individuals are asked to imagine how they would feel during those future events if they had achieved their health goals, to assist in developing cues they can recall daily, to promote health-related decision-making. As depicted in Fig. 1, EFT may facilitate a shift from engaging in immediately rewarding behaviours such as participating in sedentary activities such as watching television or consuming high-energy discretionary food to engaging in health behaviours orientated towards the future including PA and consuming core, nutrient-dense whole foods aimed at achieving long-term health goals. EFT employs multiple techniques that are described in the Behaviour Change Taxonomy, including goal and planning-related strategies as well as comparing future goals with existing behaviours^(35)^.

Experimental studies have reported promising effects of EFT across the domains of substance use and health; however, current evidence is limited to pilot-scale studies delivered in acute settings mostly outside of the health domain. For example, EFT has been shown to reduce not only future discounting, but health behaviours including cigarette smoking^(36)^, demand for alcohol in healthy samples and in patients with alcohol dependence^(37,38)^, and high-energy food intake in an *ad libitum* taste test (about 300 calories) in people with obesity^(39)^. In a recent systematic review of future discounting interventions^(30)^, only two out of ninety-eight studies focused on diet and PA, with most studies focused on financial behaviours/spending or substance use, such as cannabis use^(40)^. Of note, most studies have delivered acute EFT training comprising single sessions conducted in laboratory settings. A smaller subset of studies included clinically relevant settings and outcomes to promote real-life applicability. For example, a recent study examined the use of EFT training delivered daily via a smartphone app for 1 week to modify health behaviours in individuals with excess weight^(41)^. Participants reported high motivation and engagement levels with the EFT intervention, but the study duration was insufficient to observe any clinically meaningful changes in body weight. Other studies have shown EFT can reduce the energy content of foods purchased while online grocery shopping^(42)^ and consumed in a food court^(43)^. Nevertheless, these findings suggest promising effects of EFT on real-world health behaviours.

Despite promising results of EFT, generalisability to clinical populations is understudied. Pilot work incorporating brief EFT training has been shown to reduce future discounting in people with pre-diabetes^(44,45)^, weight loss in people with obesity^(46)^ and medication adherence in breast cancer survivors^(47)^. A recent 6-month multi-component lifestyle-based weight loss programme found no additive effects of EFT on weight, HbA1c or PA in people with pre-diabetes; however EFT was administered after the behavioural programme components limiting its effectiveness^(48)^. Nevertheless, the aforementioned studies have advanced our understanding of EFT as a prime candidate for interventions targeting lifestyle behaviours in populations at risk of NCD. To date, most studies have typically focused on single-domain health behaviours; however, given that future discounting is a multi-behaviour mechanism, EFT has the potential to simultaneously improve multiple lifestyle behaviours, including diet and PA^(49)^. Recent research has examined the impact of exercise-induced changes in delay discounting on monetary rewards^(50,51)^ and temporal food choice^(52)^. Nevertheless, there is a paucity of research examining the effects of EFT on diet and physical inactivity despite unhealthy lifestyle behaviours tending to be clustered and exponentially increasing NCD risk^(53)^.

Based on the established evidence that future discounting is a predictor of modifiable lifestyle behaviours underlying NCD risk, EFT represents an important strategy that could be implemented prior to or whilst engaging in lifestyle interventions to improve health outcomes^(54)^. Furthermore, emerging evidence suggests that future discounting is a modifiable treatment target and EFT is an engaging intervention that can improve health behaviours. Nevertheless, further studies are needed to evaluate the long-term effects of EFT among individuals with elevated NCD risk.

## Promising future discounting techniques for non-communicable disease prevention and management

While future discounting is a modifiable mechanism and EFT is an effective disruptive approach, such strategies have not been implemented in longer-term, community-based health interventions targeting lifestyle-related behaviours and related NCD. Indeed, existing interventions used in public health practice for NCD risk reduction or management often do not reflect our emerging understanding of psychological factors driving lifestyle behaviours and NCD risk and related cognitive training techniques^(55)^. Future clinical research should integrate future discounting strategies within lifestyle interventions as an adjunct technique to enhance adherence to diet and PA interventions. Such research should consider targeting populations with higher disease risk such as individuals with pre-diabetes or those at risk of T2DM^(48)^, as well as children or older adults who typically demonstrate elevated levels of future discounting relative to young/middle-aged adults^(56)^.

Future trials should also investigate the optimal modalities for delivering EFT including intervention commencement timing, frequency and intensity to improve effectiveness^(57)^. Previous EFT studies have used a range of delivery modes (i.e. face to face and digital health technologies) and cue types (e.g. auditory, written and drawn methods)^(58)^. Indeed, recent evidence suggests that both written and illustrated EFT cues are effective, but this is yet to be confirmed in clinical trials^(59)^. Whilst there is currently a lack of consensus regarding the optimal delivery mode and cue type for EFT, co-creation methods that involve individual and community representation should be deployed to design modifiable elements (e.g. delivery mode) that are tailored for user capabilities and health inequalities to ensure training programmes promote engagement and uptake^(7)^. Moreover, exploratory work should be undertaken to understand the impact of different approaches to EFT (i.e. delivery modes and cue types) on heterogenous communities to co-create a framework and guidelines before undertaking clinical trials with these population groups.

The proposed mechanism of action underlying the therapeutic effects of future discounting interventions should be examined in future trials. The National Institute of Health’s Science of Behavior Change Network recommends a database of measurement tools that can be used to measure the mechanisms underpinning behaviour change techniques^(60)^. In this instance, changes in future discounting should be assessed via standardised assessment tools to ensure generalisability across studies. Such measures include computerised behavioural paradigms (i.e. the delay discounting task)^(61)^, as well as self-report scales (i.e. Monetary-Choice Questionnaire)^(62)^, that involve people making explicit choices between hypothetical smaller, immediate and larger, delayed rewards such as food or money, as well as the Consideration of Future Consequences scale^(15)^, which assesses individual differences in orientation towards present or future thinking^(63)^. The inclusion of such measures will provide insights into the mechanisms of action of EFT interventions, which may involve reducing the discounting rate (i.e. increasing the subjective value of future outcomes) and thereby making the future more concrete or by modulating emotion related to the future^(64,65)^.

## Conclusion

Lifestyle interventions promoting improved dietary quality, increased PA and reduced sedentary behaviours are effective for reducing NCD risk and management. Nevertheless, uptake and adherence to such interventions remains poor, due at least in part to the underpinning psychological tendency to value smaller immediate rewards over larger future rewards. Disrupting future discounting via psychological training techniques such as EFT represents a low cost, scalable and promising approach that could be incorporated within interventions targeting lifestyle factors to increase adherence and reduce NCD risk. This innovative approach links established health Behaviour Change Taxonomy with recent advances in psychological interventions that underpin key drivers of health behaviours. Undertaking a co-creation study with end users and key stakeholders will help establish the most appropriate formats and strategies for delivering engaging EFT interventions. Further research is required to understand the efficacy and applicability of EFT to improve the effectiveness of lifestyle interventions to reduce risk and improve management of lifestyle-related chronic diseases.
